# Influenza: Can we cope better with the unpredictable?

**DOI:** 10.1080/21645515.2015.1086047

**Published:** 2015-09-11

**Authors:** Gaël Dos Santos, Elisabeth Neumeier, Rafik Bekkat-Berkani

**Affiliations:** a Business & Decision Life Sciences (on behalf of GSK Vaccines), Brussels, Belgium; b GSK Vaccines, Dresden, Germany; c GSK Vaccines, Wavre, Belgium

**Keywords:** circulating strains, influenza, vaccine-preventable diseases, vaccines and immunisation

## Abstract

Seasonal influenza vaccines are unique because they are regularly reformulated and prepared in anticipation of the upcoming influenza season. Selection of vaccine strains occurs in advance of the influenza season, allowing time for vaccine production. Influenza viruses constantly evolve, and mismatches between vaccine strains and circulating strains have occurred in the past, impacting on vaccine effectiveness. The public health impact of a mismatch depends on multiple factors including strain virulence and transmission dynamics, pre-existing population immunity to the drift strain, and cross-reactivity induced by vaccination. Influenza vaccine effectiveness thus varies unpredictably from year to year, and may differ across European and northern American regions. Here we highlight the unpredictability associated with influenza virus circulation and present a comprehensive picture of circulating influenza strains in the northern hemisphere as compared to WHO recommendations for vaccine strains over the last 15 y. In years when vaccine mismatch occurs, such as the 2014–15 influenza season, public health agencies continue to recommend influenza vaccination as the preferred means by which to protect against influenza and influenza-associated complications. Research is on-going to optimise strain selection and vaccine composition to improve effectiveness.

## Introduction

According to the World Health Organization (WHO), 5% to 15% of the adult population are affected with upper respiratory tract infections during annual influenza epidemics.^1^ The WHO estimates that epidemics caused by influenza A and B viruses cause annually between 3 and 5 million cases of severe illness, and between 250,000 and 500,000 deaths. Annual influenza vaccination is considered one of the most efficient measures to prevent influenza illness or avert its severe outcomes.^2^ This paper highlights the unpredictability associated with influenza virus circulation, which complicates annual vaccine strain selection. We provide a comprehensive picture of circulating influenza strains in the northern hemisphere using surveillance data issued by the United States Centers for Disease Control and Prevention, Public Health Agency of Canada, and the European Center for Disease Protection and Control as compared to recommendations provided by the WHO for vaccine strains selection over the last 15 y. We underscore initiatives to better predict which viruses are most likely to circulate during an up-coming influenza season, and ongoing research to improve the effectiveness of influenza vaccines. Because data in the southern hemisphere were not consistently and comprehensively reported over the last 15 years, only data derived from the northern hemisphere are presented hereafter.

In addition to 2 subtypes of influenza A virus (A/H1N1 and A/H3N2), 2 lineages of influenza B viruses (Victoria and Yamagata) typically co-circulate in humans and cause seasonal influenza epidemics.

Influenza viruses undergo constant mutation. Minor changes in the haemagglutinin antigen (HA) of influenza A strains (antigenic drift) enable the virus to cause repeated influenza outbreaks by evading immune recognition. Continual change regularly exposes populations to new, but related influenza variants each season, resulting in reduced immunity to the HA head domain.^3^ Conversely, major changes in the influenza type A surface antigens (antigenic shift) are caused by genetic reassortment between different influenza A subtypes, such as between animal/avian and human subtypes. Rarely, such shifted viruses can result in strains capable of causing large regional or global pandemic outbreaks.

Emergence of antigenic variants through antigenic drift is the virological basis that necessitates consideration for adjustment of vaccine viruses, and which makes influenza vaccines unique in that they are regularly reformulated and prepared in anticipation of the upcoming influenza season. It is noteworthy that antigenic drift may occur during the time between vaccine virus selection and the influenza season. Predicting which strains are most likely to circulate in the upcoming season (northern and southern hemispheres), is a task undertaken bi-annually by WHO using a global surveillance and response system.^4,5^ Currently, most influenza virus vaccines consist of a combination of 3 or 4 seasonal virus strains, i.e., 2 human influenza A strains (A/H3N2 and A/H1N1) and one or 2 influenza B viruses (Yamagata and/or Victoria lineage). Vaccine mismatches can occur when the antigenic characteristics of one or more of the circulating strains substantially diverge from the corresponding vaccine strains.

Seasonal influenza vaccine effectiveness (VE) is regularly measured in “real world” conditions in order to assess the vaccine's ability to protect against the prevailing influenza viruses. In seasons where a mismatch occurs, the vaccine's ability to protect against the antigenically dissimilar circulating strain may be reduced, although some cross-reactivity (against different variants) has been observed in randomized trials (both for H1N1, H3N2 drifted viruses and B lineage mismatched viruses), potentially resulting in milder illness if vaccinated individuals do become infected.^6^ Assessment of VE is therefore complicated by the degree of match between vaccine virus and circulating virus, as well as when both influenza B virus lineages co-criculate. VE thus varies unpredictably from year to year.

In the United States (US) and Canada, influenza A/H3N2 strains have substantially circulated in 9 of the last 15 seasons, although these seasons were not always the same in either country. Important A/H3N2 vaccine strain mismatches (accounting for at least 40% of A strains subtyped) observed in 4 seasons (Tables 1, 2).^7-9^ Available VE estimates for the US for these mismatched seasons were 19% in 2014-15, 37% in 2007-08 (there was also a type B mismatch during this season), and 10% in 2004-05.^10^ Additionally, B-lineage mismatches between the vaccine strain(s) and circulating strain(s) (accounting for at least 40% of B strains characterized) occurred in 6 out of 15 seasons in the US and in 7 out of 15 seasons in Canada, mainly because B viruses causing infection belonged to a genetic lineage other than the one included in the vaccine. Available US estimates of VE during seasons of influenza type B mismatch were 47% in 2011-12, 37% in 2007-08 (co-present with an A/H3N2 mismatch) and 21% in 2005-06.^10^ By contrast, VE estimates for years without mismatch ranged between 49% and 60%.^10^ A/H1N1 viruses undergo slower mutation and, vaccine mismatch only occurred with widespread circulation of the A/California/7/2009 strain in 2009-10 (and also in 2008-09 in Canada) that caused the 2009/10 H1N1 pandemic.

**Table 1.** **United States:** Antigenic match between seasonal trivalent influenza vaccine strains and circulating influenza viruses from 2000-01 to 2014-15.*^7,8^

	**TIV composition: northern hemisphere^5^**			**Circulating viruses: United States**					
				**% of all positive specimens**		**% of Type A (with subtyping performed)***predominant strain (with characterisation performed)*		**% of Type B lineages***predominant strain (with characterisation performed)*	
**Season**	**A(H1N1)**	**A(H3N2)**	**Type B**	**A**	**B**	**A/H1N1**	**A/H3N2**	**Yamagata**	**Victoria**
2014-15	A/California/7/2009pdm09	A/Texas/50/2012	B/Massachusetts/2/2012 (YAM)†	83.5%	16.5%	**0.4%**	**99.6%**	**71.9%**	**28.1%**
(week 20)						*A/California/7/2009 (100%)*	*A/Switzerland/9715293/201 (predominant)*	*B/Massachusetts/2/2012 (98.1%)*	*B/Brisbane/60/2008 (97.8%)*
							*A/Texas/50/2012 (18.6%)*		
2013-14	A/California/7/2009pdm09	A/Texas/50/2012	B/Massachusetts/2/2012 (YAM)†	87.4%	12.6%	**90.3%**	**9.7%**	**72.9%**	**27.1%**
						*A/California/7/2009 (99.9%*)	*A/Texas/50/2012 (95.3%)*	*B/Massachusetts/2/2012 (99.0%)*	*B/Brisbane/60/2008 (100%)*
2012-13	A/California/7/2009pdm09	A/Victoria/361/2011	B/Wisconsin/1/2010 (YAM)	70.0%	30.0%	**6.0%**	**94.0%**	**63.8%**	**36.2%**
						*A/California/7/2009 (98.7%)*	*A/Victoria/361/2011 (99.6%)*	*B/Wisconsin/1/2010 (100%)*	*Not specified*
2011-12	A/California/7/2009	A/Perth/16/2009	B/Brisbane/60/2008 (VIC)	82.0%	18.0%	**25.0%**	**73.0%**	**48.0%**	**52.0%**
						*A/California/7/2009 (96.0%)*	*A/Perth/16/2009 (75.0%)*	*B/Wisconsin/01/2010 (100%)*	*B/Brisbane/60/2008 (95.0%)*
							*A/Victoria/361/2011 (3.0%)*		
2010-11	A/California/7/2009	A/Perth/16/2009	B/Brisbane/60/2008 (VIC)	74.0%	26.0%	**38.0%**	**62.0%**	**5.8%**	**94.0%**
						*A/California/7/2009 (99.8%)*	*A/Perth/16/2009 (96.8%)*	*Not specified*	*B/Brisbane/60/2008 (99.9%)*
2009-10	A/Brisbane/59/2007	A/Brisbane/10/2007	B/Brisbane/60/2008 (VIC)	99.0%	1.0%	**95.0%**	**4.0%**	**11.6%**	**88.4%**
						*A/California/7/2009 (99.5%)*	*A/Perth/16/2009 (100%)*	*Not specified*	*B/Brisbane/60/2008 (100%)*
2008-09	A/Brisbane/59/2007	A/Brisbane/10/2007	B/Florida/4/2006 (YAM)	66.0%	34.0%	**87.0%**	**13.0%**	**17.0%**	**83.0%**
						*A/Brisbane/59/2007 (100%)*	*A/Brisbane/10/2007 (100%)*	*B/Florida/04/2006 (100%)*	*Not specified*
2007-08	A/Solomon Islands/3/2006	A/Wisconsin/67/2005	B/Malaysia/2506/2004 (VIC)	71.0%	29.0%	**26.0%**	**74.0%**	**98.0%**	**2.0%**
						*A/Solomon Islands/3/2006 (66.0%)*	*A/Brisbane/10/2007 (60.0%) A/Wisconsin/67/2005 (23.0%)*	*B/Florida/4/2006 (89.0%)*	B/Ohio/01/2005** (75.0%)
						*A/Brisbane/59/2007 (29.0%)*			
2006-07	A/New Caledonia/20/99	A/Wisconsin/67/2005	B/Malaysia/2506/2004 (VIC)	79.2%	20.8%	**62.3%**	**37.7%**	**23.0%**	**77.0%**
						*A/New Caledonia/20/99 (90.0%)*	*A/Wisconsin/67/2005 (24.0%)*	*Not specified*	B/Ohio/01/2005** (50.0%)
2005-06	A/New Caledonia/20/99	A/California/7/2004	B/Shanghai/361/2002 (YAM)	79.7%	20.3%	**8.1%**	**91.9%**	**18.7%**	**81.3%**
						*A/New Caledonia/20/99 (97.0%)*	*A/California/07/2004 (72.8%) A/Wisconsin/67/2005*	*B/Florida/07/2004 (81.7%)B/Shanghai/361/2002 (16.7%)*	*B/Ohio/1/2005 (99.9%)*
									
2004-05	A/New Caledonia/20/99	A/Fujian/411/2002	B/Shanghai/361/2002 (YAM)	75.4%	24.6%	**0.3%**	**99.7%**	**74.4%**	**25.6%**
						*A/New Caledonia/20/99 (100%)*	A/California/7/2004 (78.0%) A/Wyoming/3/2003** (22.0%)	*B/Shanghai/361/2002 (83.0%)*	*Not specified*
2003-04	A/New Caledonia/20/99	A/Moscow/10/99	B/HongKong/330/2001 (VIC)	99.0%	1.0%	**0.1%**	**99.9%**	**93.0%**	**7.0%**
						*Not specified*	*A/Fujian/411/2002 (H3N2) (88.8%)*	*B/Sichuan/379/99 (100%)*	*B/Hong Kong/330/2001 (100%)*
							A/Panama/2007/99** (11.2%)		
2002-03	A/New Caledonia/20/99	A/Moscow/10/99	B/HongKong/330/2001 (VIC)	56.4%	43.6%	**70.3%**	**29.7%**	**0.4%**	**99.6%**
						*A/New Caledonia/20/99 (100%)*	A/Panama/2007/99** (85.0%)	*B/Shizuoka/15/2001*	*B/Hong Kong/330/01*
2001-02	A/New Caledonia/20/99	A/Moscow/10/99	B/Sichuan/379/99 (YAM)	87.5%	12.5%	**1.9%**	**98.1%**	**22.8%**	**77.2%**
						*A/New Caledonia/20/99 (100%)*	A/Panama/2007/99** (100%)	*B/Sichuan/379/99 (21.3%)*	*Not specified*
								*B/Shizuoka/15/2001 (predominant)*	
2000-01	A/New Caledonia/20/99	A/Moscow/10/99	B/Beijing/184/93 (YAM)	54.0%	46.0%	**97.0%**	**3.0%**	**100%**	
						*A/New Caledonia/20/99 (95.0%)*	A/Panama/2007/99** (100%)	*B/Beijing/184/93 (11.0%)*	
						*A/Bayern/07/95 (5.0%)*		B/Sichuan/379/99** (89.0%)	

* For influenza A, mismatch accounting for at least 40% of A-viruses antigenically characterised are indicated in yellow shading. For influenza B, co-circulation or mismatch accounting for at least 40% of B-viruses antigenically characterised are indicated in dark shading

**Viruses characterised that were antigenically similar to the strains specified by the WHO, for example A/Panama/2007/99 is an A/Moscow/10/99 (H3N2)-like virus.

†As from 2013-14 WHO recommends that quadrivalent seasonal influenza vaccines contain a second influenza B virus strain: in 2013-14 and 2014-15 this was a B/Brisbane/60/2008-like virus (VIC)

YAM = Yamagata lineage, VIC = Victoria lineage.

**Table 2.** **Canada:** Antigenic match (yellow shading) between seasonal trivalent influenza vaccine strains and circulating influenza viruses from 2000-01 to 2014-15.*^9^

	**TIV composition: northern hemisphere ^5^**			**Circulating viruses: Canada**					
				**% of all positive specimens**		**% of Type A (with subtyping performed)***predominant strain (with characterisation performed)*		**% of Type B lineages***predominant strain (with characterisation performed)*	
**Season**	**A(H1N1)**	**A(H3N2)**	**Type B**	**Influenza A**	**Influenza B**	**A/H1N1**	**A/H3N2**	**Yamagata**	**Victoria**
2014-15	A/California/7/2009pdm09	A/Texas/50/2012	B/Massachusetts/2/2012 (YAM)†	80.6%	19.4%	**0.8%**	**99.2%**	**92.5%**	**7.5%**
(week 20)						*A/California/7/2009 (100%)*	*A/Switzerland/9715293/2013 **(97.0%)***	*B/Massachusetts/2/2012 (99.6%)*	*B/Brisbane/60/2008 (100%)*
							*A/Texas/50/2012 (0.5%)*		
2013-14	A/California/7/2009pdm09	A/Texas/50/2012	B/Massachusetts/2/2012 (YAM)†	73.7%	26.3%	**94.0%**	**6.0%**	**96.6%**	**3.4%**
						*A/California/7/2009 (100%)*	*A/Texas/50/2012 (99.0%)*	*B/Massachusetts/2/2012 (100%)*	*B/Brisbane/60/2008 (100%)*
2012-13	A/California/7/2009pdm09	A/Victoria/361/2011	B/Wisconsin/1/2010 (YAM)	85%	15%	**12.0%**	**88.0%**	**77.0%**	**23%**
						*A/California/7/2009 (100%)*	*A/Victoria/361/2011 (100%)*	*B/Wisconsin/1/2010 (100%)*	*B/Brisbane/60/2008 (100%)*
2011-12	A/California/7/2009	A/Perth/16/2009	B/Brisbane/60/2008 (VIC)	47%	53%	**31.0%**	**69.0%**	**52.3%**	**47.7%**
						*A/California/7/2009 (97.7%)*	*A/Perth/16/2009 (91.4%)*	*B/Wisconsin/01/2010 (100%)*	*B/Brisbane/60/2008 (100%)*
2010-11	A/California/7/2009	A/Perth/16/2009	B/Brisbane/60/2008 (VIC)	84.5%	15.5%	**14.0%**	**86.0%**	**4.9%**	**95.1%**
						*A/California/7/2009 (98.7%)*	*A/Perth/16/2009 (98.9%)*	*B/Wisconsin/01/2010 (100%)*	*B/Brisbane/60/2008 (100%)*
2009-10	A/Brisbane/59/2007	A/Brisbane/10/2007	B/Brisbane/60/2008 (VIC)	99.9%	0.1%	**99.9%**	**0.2%**	**14.3%**	**85.7%**
						*A/California/7/2009 (99.7%)*	*A/Perth/16/2009 (80.0%)*	*B/Florida/4/2006*	*B/Brisbane/60/2008 (71.4%)*
						*A/Brisbane/59/2007 (0.4%)*	*A/Brisbane/10/2007 (20.0%)*		*B/Malaysia/2506/2004 (14.3%)*
2008-09	A/Brisbane/59/2007	A/Brisbane/10/2007	B/Florida/4/2006 (YAM)	83.3%	16.7%	**96.0%**	**4.0%**	**1.9%**	**98.1%**
						*A/Brisbane/59/2007 (43.9%)*	*A/Brisbane/10/2007 (100%)*	*B/Florida/4/2006 (100%)*	*B\/Malaysia/2506/2004 (66.5%)*
						*A/California/07/2009 (56.1)*			*B/Brisbane/60/2008 (31.6%)*
2007-08	A/Solomon Islands/3/2006	A/Wisconsin/67/2005	B/Malaysia/2506/2004 (VIC)	57.5%	42.5%	**35.9%**	**64.1%**	**97.5%**	**2.5%**
						*A/Solomon Islands/3/2006 (94.8%)*	*A/Brisbane/10/2007 (94.3%)*	*B/Florida/4/2006 (100%)*	*B/Malaysia/2506/2004 (100%)*
						*A/Brisbane/59/2007 (5.2%)*	*A/Wisconsin/67/2005 (5.7%)*		
2006-07	A/New Caledonia/20/99	A/Wisconsin/67/2005	B/Malaysia/2506/2004 (VIC)	86.2%	13.8%	**27.0%**	**61.4%**	**89.9%**	**10.1%**
						*A/New Caledonia/20/99 (100%)*	*A/Wisconsin/67/2005 (85.4%)*	*B/Shanghai/361/2002 (100%)*	*B/Malaysia/2506/2004 (100%)*
2005-06	A/New Caledonia/20/99	A/California/7/2004	B/Shanghai/361/2002 (YAM)	61.1%	38.9%	**9.6%**	**44.6%**	**1.5%**	**98.5%**
						*A/New Caledonia/20/99 (100%)*	*A/California/07/2004 (87.6%)*	*B/Shanghai/361/2002 (100%)*	*B/Malaysia/2506/2004 (70.8%)*
							*A/Wisconsin/67/2005 (12.4%)*		*B/Hong Kong/330/2001 (29.2%)*
2004-05	A/New Caledonia/20/99	A/Fujian/411/2002	B/Shanghai/361/2002 (YAM)	83.6%	16.4%	**1.0%**	**99.0%**	**79.0%**	**21.0%**
						*Not specified*	*A/Fujian/411/02 (57.0%)*	*B/Shanghai/361/2002 (100%)*	*B/Hong Kong/ 330/01 (100%)*
							*A/California/7/2004 (43.0%)*		
2003-04	A/New Caledonia/20/99	A/Moscow/10/99	B/Hong Kong/330/2001 (VIC)	98.7%	1.2%	**0.4%**	**94.8%**	**82.5%**	**17.5%**
						*A/New Caledonia/20/99(100%)*	*A/Fujian/411/2002 (96.8%)*	*B/Sichuan/379/99 (100%)*	*B/Hong Kong/330/01 (100%)*
							A/Panama/2007/99** (3.1%)		
2002-03	A/New Caledonia/20/99	A/Moscow/10/99	B/Hong Kong/330/2001 (VIC)	58.0%	42.0%	**23.0%**	**16.0%**	*Not specified*	*B/Hong Kong/330/01 (predominant)*
						*A/New Caledonia/20/99 [77% H1N2] §*	A/Panama/2007/99**(100%)		
2001-02	A/New Caledonia/20/99	A/Moscow/10/99	B/Sichuan/379/99 (YAM)	87.0%	13.0%	**0.2%**	**82.0%**	**3.3%**	**96.7%**
						*A/New Caledonia/20/99 [17.7% H1N2] §*	A/Panama/2007/99** (100%)	*B/Sichuan/379/99 (100%)*	*B/Hong Kong/330/01 (100%)*
2000-01	A/New Caledonia/20/99	A/Moscow/10/99	B/Beijing/184/93 (YAM)	32.0%	68.0%	**99.4%**	**0.6%**	**99.6%**	**0.4%**
						*A/New Caledonia/20/99 (97.0%) A/Johannesburg/82/96 (1.0%)*	A/Panama/2007/99** (100%)	B/Yamanashi/166/98** (100%)	*B/Beijing/243/97(100%)*

* For influenza A, mismatch accounting for at least 40% of A-viruses antigenically characterised are indicated in yellow shading. For influenza B, co-circulation or mismatch accounting for at least 40% of B-viruses antigenically characterised are indicated in dark shading

**Viruses characterised that were antigenically similar to the strains specified by the WHO, for example A/Panama/2007/99 is an A/Moscow/10/99 (H3N2)-like virus.

†As from 2013-14 WHO recommends that quadrivalent seasonal influenza vaccines contain a second influenza B virus strain: in 2013-14 and 2014-15 this was a B/Brisbane/60/2008-like virus (VIC)

YAM = Yamagata lineage, VIC = Victoria lineage.

§ The H1 antigen was identical to the vaccine strain

In Europe, A/H3N2 strains have significantly circulated (>50% of A strains) in 8 out of the last 15 influenza seasons, with A/H3N2 vaccine mismatches observed in 3 seasons, and an H1N1 mismatch in 2009-10 (Table 3: Note that information in Table 3 is derived from European Union (EU) reports which summarise data from different countries across which virus circulation may have been heterogeneous).^11^ In addition, B-lineage mismatches occurred in 4 out of 15 seasons. It is noteworthy that while the same vaccine strains are used in seasonal influenza vaccines distributed in US, Canada, and Europe, the strains circulating and causing disease in those regions may differ significantly, resulting in heterogeneous VE.

**Table 3.** **Europe:** Antigenic match between seasonal trivalent influenza vaccine strains and circulating influenza viruses from 2000-01 to 2014-15.*^11^

	TIV composition: northern hemisphere ^5^			Circulating viruses: Europe					
	A(H1N1)	A(H3N2)	Type B	% of all positive specimens		% of Type A (with subtyping performed) *predominant strain (with characterisation performed)*		% of Type B lineages *predominant strain (with characterisation performed)*	
Season				A	B	A/H1N1	A/H3N2	Yamagata	Victoria
2014-15	A/California/7/2009pdm09	A/Texas/50/2012	B/Massachusetts/2/2012 (YAM)†	67%	33%	**23.1%**	**76.9%**	**97.7%**	**2.3%**
(week 20)						*A/California/07/2009*	*A/Switzerland/9715293/2013 (71.0% of H3)*	*B/Massachusetts/02/2012 (19.9% of YAM)*	*B/Brisbane/60/2008 (100% of VIC)*
							*A/Texas/50/2012 (29.0% of H3)*	*B/Phuket/3073/2013 (79.2% of YAM) B/Wisconsin/1/2010 (0.1% of YAM)*	
								*B/Florida/4/2006 (0.7% of YAM)*	
									
2013-14	A/California/7/2009pdm09	A/Texas/50/2012	B/Massachusetts/2/2012 (YAM)†	98%	2%	*A/California/07/2009*	*A/Texas/50/2012*	**69.5%**	**30.5%**
						*(60.0% of all)*	*(37.0% of all)*	*B/Wisconsin/1/2010(12.2% of YAM)*	*B/Brisbane/60/2008(100% of VIC)*
								*B/Massachusetts/02/2012 (85.4% of YAM)*	
2012-13	A/California/7/2009pdm09	A/Victoria/361/2011	B/Wisconsin/1/2010 (YAM)	48.5%	51.5%	**58.0%**	**42.0%**	**86.0%**	**14.0%**
						*A/California/07/2009*	*A/Victoria/361/2011*	*B/Wisconsin/1/2010 (25.0% of all)*	*B/Brisbane/60/2008*
						*(9.5% of all)*	*(55.0% of all)*	*B/Florida/4/2006 (0.5% of all)*	*(6.6% of all)*
2011-12	A/California/7/2009	A/Perth/16/2009	B/Brisbane/60/2008 (VIC)	89.3%	10.7%	**1.5%**	**98.5%**	**39.6%**	**60.4%**
						*A/California/07/2009*	*A/Perth/16/2009 (74.3%)*	*not specified*	*not specified*
							*(58.1% of A/H3 viruses had an imperfect genetic match with vaccine strain)*		
2010-11	A/California/7/2009	A/Perth/16/2009	B/Brisbane/60/2008 (VIC)	59.6%	40.4%	**97.0%**	**3.0%**	**<10%**	*B/Brisbane/60/20089 *
						*A/California/07/2009*	*A/Perth/16/2009*	*B/Florida/4/2006*	*(Predominant)*
								*B/Bangladesh/3333/2007*	
2009-10	A/Brisbane/59/2007	A/Brisbane/10/2007	B/Brisbane/60/2008 (VIC)	99%	1%	*A/California/07/2009 (99.7% of all)*	<*0.1% of all*	*Not specified*	*Not specified*
									
2008-09	A/Brisbane/59/2007	A/Brisbane/10/2007	B/Florida/4/2006 (YAM)	81.4%	18.6%	*A/Brisbane/59/2007 (3.7% of all)*	*A/Brisbane/10/2007 (37.1% of all)*	*B/Florida/4/2006 (0.8% of all)*	*B/Malaysia/2506/2004 (20.7% of all)*
									*B/Brisbane/60/2008 (1.6% of all)*
									
2007-08	A/Solomon Islands/3/2006	A/Wisconsin/67/2005	B/Malaysia/2506/2004 (VIC)	60%	40%	*A/Solomon Islands/3/2006 (58.0% of all)*	*A/Brisbane/10/2007 (1.5% of all)*	*B/Florida/4/2006 predominant (38.0% of all)*	*B/Malaysia/2506/2004 (0.5% of all)*
						*A/New Caledonia/20/99 (1.5% of all)*	*A/Wisconsin/67/2005 (0.8% of all)*		
2006-07	A/New Caledonia/20/99	A/Wisconsin/67/2005	B/Malaysia/2506/2004 (VIC)	97.2%	2.8%	**10.7%**	**89.3%**	*B/Jiangsu/10/2003 (1.0% of all)*	*B/Malaysia/2506/2004 (4.0% of all)*
						*A/New Caledonia/20/99 (8.0% of all)*	*A/Wisconsin/67/2005 (86.0% of all)*		
							*A/California/7/2004 (1.0% of all)*		
2005-06	A/New Caledonia/20/99	A/California/7/2004	B/Shanghai/361/2002 (YAM)	42.0%	58.0%	**48.0%**	**51.8%**	*Not specified*	*Not specified*
						*Not specified*	*Not specified*		
2004-05	A/New Caledonia/20/99	A/Fujian/411/2002	B/Shanghai/361/2002 (YAM)	83.3%	16.7%	**18.2%**	**81.8%**	*Not specified*	**43.0%**
						*Not specified*	*A/Wellington/1/2004*		*B/Hong Kong/330/2001*
							*A/California/7/2004*		
2003-04	A/New Caledonia/20/99	A/Moscow/10/99	B/HongKong/330/2001 (VIC)	99.1%	0.9%	**0.5%**	**99.1%**	*Not specified*	*Not specified*
						*Not specified*	*Not specified*		
2002-03	A/New Caledonia/20/99	A/Moscow/10/99	B/HongKong/330/2001 (VIC)	63.4%	36.4%	**9.7%**	**88.8%**	*Not specified*	*Not specified*
						*Not specified*	*Not specified*		
2001-02	A/New Caledonia/20/99	A/Moscow/10/99	B/Sichuan/379/99 (YAM)	74.9%	25.1%	**3.8%**	**87.3%**	*Not specified*	*Not specified*
						*Not specified*	*Not specified*		
2000-01	A/New Caledonia/20/99	A/Moscow/10/99	B/Beijing/184/93 (YAM)	70.3%	29.7%	**96.7%**	**3.1%**	*Not specified*	*Not specified*
						*Not specified*	*Not specified*		

* For influenza A, mismatch accounting for at least 40% of A-viruses antigenically characterised are indicated in yellow shading. For influenza B, co-circulation or mismatch accounting for at least 40% of B-viruses antigenically characterised are indicated in dark shading

†As from 2013-14 WHO recommends that quadrivalent seasonal influenza vaccines contain a second influenza B virus strain: in 2013-14 and 2014-15 this was a B/Brisbane/60/2008-like virus (VIC)

YAM = Yamagata lineage, VIC = Victoria lineage.

(X% of all) = percentage of all viruses (A +B) that were antigenically subtyped

% of A/B etc = percentage of all viruses subtyped as ‘A’ or ‘B’ etc

Note that numbers were derived from EU reports which summarise data from the different EU countries in which the virus circulation was not always perfectly homogenous.

The implications of a vaccine mismatch or co-circulation of both influenza B virus lineages on the clinical presentation of subjects infected with influenza illness are difficult to foresee. The public health impact depends on multiple factors such as strain virulence, transmission dynamics, pre-existing population immunity to the drift strain, the age-groups most prone to infection, the extent of the geographic distribution of the drifted strain, and characteristics (immunogenicity) of the vaccine itself.^6^ A mismatch in the A/H3N2 vaccine component, or co-circulation with a B-lineage mismatch, raise particular public health concerns because these scenarios are often associated with more severe influenza illnesses, hospitalisations, and deaths.^12^ VE against laboratory-confirmed influenza infection varies substantially across seasons, and is highest when the antigenic match is optimal. Modeling studies estimated that VE ranged between 10% and 60% in the US from 2005 to 2015, with an overall estimated number of deaths averted by seasonal vaccination between 222 and 9398 (2005–06 to 2013–14 seasons).^10,13^ Similar seasonal variations in VE have been observed in Europe.^14,15^

In the last (2014–15) influenza season, drifted A/H3N2 viruses were first detected in late March 2014, after WHO recommendations for the 2014–15 northern hemisphere vaccine had been issued.^16^ Characterization of the circulating influenza A/H3N2 strain viruses in Europe and North America showed similar antigenic drift from the vaccine strain in the majority of isolates, raising concern early in the season that VE against laboratory-confirmed influenza might be suboptimal.^9,17,18^ Recently published interim estimates of 2014–15 influenza VE from the US, Canada and the United Kingdom have corroborated those concerns (Table 4).^19-22^

**Table 4.** Influenza vaccine effectiveness in the 2014-15 northern hemisphere season.

Country	Outcome measure	Population	VE (95% CI)
United States ^20^	Laboratory-confirmed, medically attended acute respiratory illness	6 months -17 years	24% (0; 43)
		18-49 years	16% (−18; 41)
		≥ 50 years	23% (−14 ; 47)
United Kingdom ^21^	Primary care consultation with laboratory-confirmed influenza	All ages	3.4% (44.8; 35.5)*
	Primary care consultation with laboratory-confirmed A/H3N2 influenza	All ages	2.3% (−56.2; 33.0)*
Canada ^22^	Laboratory-confirmed, medically attended influenza	All ages	−4% (−45; 25)
	Laboratory-confirmed, medically attended A/H3N2 influenza	All ages	−8% (−50; 23)
Canada ^19^	Laboratory-confirmed influenza-related hospitalisation	≥ 16 years**	−16.8% (−48.9; 8.3)†
		< 65 years**	10.8% (−50.2; 47.0)†
		≥ 65 years**	−25.4% (−65.0; 4.6) †

*adjusted for age group, sex, month, surveillance scheme and primary school area.

**Adjusted for age and co-morbidity.

† 90% CI

Despite the low VE of the A/H3N2 component in the 2014–15 northern hemisphere influenza vaccine, the WHO, European Center for Disease Control and Prevention and US Centers for Disease Control and Prevention continued to strongly recommended influenza vaccination using 2014–15 vaccines as the preferred means by which to protect against influenza and influenza-associated complications.^18,23,24^ In the 2014/15 season, there was no mismatch between the vaccine strains and circulating strains of H1N1 and influenza B, and even though vaccination may not have fully prevented A/H3N2 infection, it may have mitigated or shortened the duration of illness and is likely to have reduced the number of hospitalisations and deaths.^24^ Modeling studies also suggest that a substantial number of hospitalisations can be averted, in particular among older adults (aged ≥65 years) even when VE is low. For instance, a US study found that a vaccine with 10% to 40% VE would avert around 13,000 to 60,000 influenza-associated hospitalisations in older adults during a moderately severe influenza season.^25^

Nevertheless, complementary strategies to control and prevent influenza transmission, such as frequent hand washing and encouraging people to stay home when sick, are particularly important in seasons during which the vaccines components are sub-optimally matched to the circulating strains. In such seasons, antiviral medications have an enhanced role to reduce the burden of disease, and are also recommended to be appropriately used to treat suspected cases of influenza, ideally within 48 hours after symptom onset, especially among individuals at high risk of influenza complications.^24^

Appropriate monitoring of influenza virus activity is another key component of the strain selection process. Using combined epidemiological, virological data and mathematical models, multiple stakeholders, including public health agencies, surveillance networks and vaccine manufacturers are actively working together in order to more accurately determine which strains are more likely to circulate during the upcoming influenza season, with the ultimate goal of deploying effective influenza vaccines each season in a timely manner.^26^

Currently available seasonal influenza vaccines require regular reformulation, and the majority are dependent on the restricted number of strains that are approved, and that can be manufactured and included in each season, the variable immunogenicity induced in different age groups and in different seasons. Manufacturers are working toward improving influenza vaccines, either by modifying existing vaccine composition, or developing new vaccine formulations that aim to provide broader protection. Modifications to the composition of seasonal influenza vaccines include the development of adjuvanted influenza vaccines that could be important in age-groups such as the elderly and young children in whom seasonal vaccines may have lower VE due to immune senescence or immaturity of the immune system. Another approach is the use of high antigen dose vaccines for which superior efficacy and higher vaccine efficacy relative to standard dose vaccines have been reported in adults aged ≥65 years treated in both community and inpatient settings.^27,28^

With the intent to increase the effectiveness of influenza vaccines, the recent availability of quadrivalent seasonal vaccines that includes influenza B strain from each of the co-circulating lineages (Yamagata and Victoria), the uncertainty related to the choice of B-lineage is overcome. Quadrivalent seasonal vaccines are therefore expected offer broader protection in seasons characterized by a lineage mismatch and/or co-circulation.^29^

There is also great interest in investigating the feasibility of a universal influenza vaccine that would be capable of inducing an immune response of sufficient breadth and depth to trigger long-term protective immunity against multiple influenza strains.^30^ Such a vaccine could result in a substantial benefit, allowing a better influenza control worldwide, particularly if effective in children and the elderly.^31^

To summarise, current seasonal influenza vaccines provide strain-specific immunity. The constantly evolving nature of influenza viruses mandates that the composition of seasonal influenza vaccines is adjusted regularly in order to remain effective. This is currently achieved through continuous global monitoring and assessment of mid-season VE estimates. Seasons characterized by low VE, such as the 2014–15 influenza season, highlight the need to continuously use advanced tools and innovative approaches to allow accurate strain selection and improved vaccine formulations in order to provide more stable VE across seasons. Although not perfect, influenza vaccination remains the main prevention strategy recommended by health agencies/authorities given that influenza continues to have a major worldwide impact, causing significant human suffering and economic burden. Accordingly, supranational organizations recently reaffirmed their recommendation for annual influenza vaccination for the 2015–16 season.

## Abbreviations

*EU*: European Union
*HA*: haemagglutinin antigen
*US*: United States
*VE*: vaccine effectiveness
*WHO*: World Health Organization
